# Development and Characterization of Lyophilized Chondroitin Sulfate-Loaded Solid Lipid Nanoparticles: Encapsulation Efficiency and Stability

**DOI:** 10.3390/pharmaceutics17010086

**Published:** 2025-01-10

**Authors:** Marta E. Bustos Araya, Anna Nardi Ricart, Ana C. Calpena Campmany, Rafel Prohens, Montserrat Miñarro Carmona

**Affiliations:** 1 Instituto de Investigaciones Farmacéuticas, Facultad de Farmacia, Universidad de Costa Rica, San José 11501, Costa Rica; marta.bustosaraya@ucr.ac.cr; 2 Pharmacy, Pharmaceutical Technology and Physico-Chemical Department, University of Barcelona, Av. Joan XXIII, 27-31, 08028 Barcelona, Spain; anacalpena@ub.edu (A.C.C.C.); minarromontse@ub.edu (M.M.C.); 3 Institute of Nanoscience and Nanotechnology (IN2UB), University of Barcelona, 08028 Barcelona, Spain; 4 Laboratory of Organic Chemistry, Faculty of Pharmacy and Food Sciences, University of Barcelona, Av. Joan XXIII, 27-31, 08028 Barcelona, Spain; rafel_prohens@ub.edu; 5 IDIBELL-UB Research Group, Pharmacotherapy, Pharmacogenomics and Pharmaceutical Technology, Avinguda Granvia, 199-203, L’Hospitalet de Llobregat, 08908 Barcelona, Spain

**Keywords:** solid lipid nanoparticles, chondroitin sulfate, lyophilization, stability studies, encapsulation efficiency

## Abstract

This study explores the development and characterization of lyophilized chondroitin sulfate (CHON)-loaded solid lipid nanoparticles (SLN) as an innovative platform for advanced drug delivery. **Background/Objectives:** Solid lipid nanoparticles are increasingly recognized for their biocompatibility, their ability to encapsulate diverse compounds, their capacity to enhance drug stability, their bioavailability, and their therapeutic efficacy. **Methods:** CHON, a naturally occurring glycosaminoglycan with anti-inflammatory and regenerative properties, was integrated into SLN formulations using the hot microemulsion technique. Two formulations (SLN-1 and SLN-2) were produced and optimized by evaluating critical physicochemical properties such as particle size, zeta potential, encapsulation efficiency (EE%), and stability. The lyophilization process, with the addition of various cryoprotectants, revealed trehalose to be the most effective agent in maintaining nanoparticle integrity and functional properties. **Results:** Morphological analyses using transmission electron microscopy (TEM) and atomic force microscopy (AFM) confirmed the dimensions of the nanoscales and their structural uniformity. Differential scanning calorimetry (DSC) and X-ray diffraction (XRD) revealed minimal excipient interaction with CHON, ensuring formulation stability. Stability studies under different environmental conditions highlighted that SLN-2 is the most stable formulation, maintaining superior encapsulation efficiency (≥88%) and particle size consistency over time. **Conclusions:** These findings underscore the potential of CHON-loaded SLNs as promising candidates for targeted, sustained-release therapies in the treatment of inflammatory and degenerative diseases.

## 1. Introduction

In recent years, nanoparticle-based drug delivery systems have drawn considerable attention because of their capacity to enhance therapeutic efficacy and bioavailability. Among these systems, solid lipid nanoparticles (SLN) stand out due to their biocompatibility, their ability to encapsulate both hydrophilic and hydrophobic compounds, and their potential to improve drug stability [1,2,3]. SLN offer distinct advantages, such as protecting active pharmaceutical ingredients, controlled drug release, targeted delivery, reduced toxicity, and increased penetration upon topical application, which collectively make them highly versatile in a range of biomedical applications [4,5].

Chondroitin sulfate (CHON), a naturally occurring glycosaminoglycan derived from various animal sources, has become a significant biomolecule in the field of drug delivery, recognized for its roles in cellular signaling and tissue repair [6,7]. The integration of CHON into SLN provides a promising approach to maximizing the therapeutic potential of nanoparticles, especially in addressing inflammatory and degenerative conditions [8,9]. The unique structural properties of CHON, when combined with the adaptability of SLN, facilitate the creation of formulations capable of improved drug entrapment, enhanced stability, and controlled release mechanisms [10].

This study aims at developing and analyzing CHON-based SLN through the hot microemulsion technique, evaluating critical physicochemical properties such as encapsulation efficiency, particle size, and zeta potential [5,11]. Additionally, the effects of different cryoprotectants on the stability of lyophilized CHON-SLN will be assessed, as studies show that lyophilization can extend nanoparticle shelf life and maintain functional properties by preventing aggregation and degradation [12,13]. Through detailed morphological and thermal characterization, the study seeks to elucidate interactions between CHON and the lipid matrix, thereby optimizing formulation stability and functionality [14,15]. Lyophilization, or freeze-drying, is a key technique employed to extend the shelf life and maintain the structural integrity of nanoparticle formulations. This process is particularly advantageous for nanoparticles containing sensitive molecules like CHON as it mitigates aggregation and preserves bioactivity and physical stability [16,17]. These stability aspects are critical for developing drug delivery systems intended for chronic conditions, in which consistent dosing is essential [18,19]. Cryoprotectants are widely employed during the freeze-drying (lyophilization) process to safeguard nanoparticles from damage caused by ice crystal formation, which can physically alter the particles and affect their properties. During the freezing phase of lyophilization, the water in the formulation freezes and forms ice crystals. If the nanoparticles are not adequately protected, this ice formation can result in the collapse or aggregation of nanoparticles. Examples of these substances include trehalose, glucose, mannose, maltose, lactose, sorbitol, mannitol, glycine, polyvinylpyrrolidone (PVP), polyvinyl alcohol (PVA), and gelatine [20].

Ensuring that nanoparticles are stable is central to their effectiveness in drug delivery applications. This includes physicochemical stability, which encompasses structural integrity, particle size consistency, and active ingredient retention over time [21,22]. In this study, CHON-loaded SLN will be subjected to stability testing under conditions compliant with the International Conference on Harmonisation (ICH) guidelines, assessing parameters like temperature, humidity, and duration [23,24]. Such analyses are indispensable to identify optimal storage conditions that maintain the bioactive properties and controlled release capacities of the nanoparticles, thereby maximizing their therapeutic potential [25].

In summary, this study advances the development of a CHON-based SLN delivery system with an emphasis on stability and controlled release. By making the most of the anti-inflammatory and regenerative properties of CHON alongside the structural benefits of SLN, this topically administered formulation offers significant promise for targeted, sustained-release therapies in the treatment of inflammatory and degenerative diseases [26,27]. This research work contributes a foundational approach to nanomedicine, with implications for expanding therapeutic options for conditions requiring extended, localized intervention [28,29].

## 2. Materials and Methods

### 2.1. Materials

The components used for nanoparticles manufacturing are octadecylamine (Acros Organics, Geel, Belgium), chondroitin sulfate (batch: F-042903) (Bioibérica, Industrial Estate, Barcelona, Spain), stearic acid 50 vegetable grade (Merck, Darmstadt, Germany), purified water (Milli-Q A10 module, Millipore, Guyancourt, France), poloxamer 188 (Sigma-Aldrich by Merck, Darmstadt, Germany), and qualitative paper filters of 43–48 μm and 7–9 μm pore size (Filter-Lab^®^, Filtros Anoia, SA, Barcelona, Spain). The components used for the lyophilization process are trehalose (Cargill-Cerestar, Indianapolis, IN, USA), trehalose (Panreac, Barcelona, Spain), mannitol (Merck, Darmstadt, Germany), povidone K30 (Thermo Scientific, Barcelona, Spain), glycine (Merck, Darmstadt, Germany), sodium dihydrogen phosphate anhydrous (Merck, Darmstadt, Germany) and sodium hydrogen diphosphate anhydrous (Merck, Darmstadt, Germany)

The reagents used for the characterization of the nanoparticles are uranyl acetate solution (Sigma Aldrich, Darmstadt, Germany), 1-cetylpyridinium chloride monohydrate (Alfa Aesar by Thermo Fisher Scientific, Bremen, Germany), NaOH (Merck, Darmstadt, Germany), sodium tetraborate decahydrate (Sigma-Aldrich by Merck, Saint Louis, MO, USA), carbazole solution (Sigma-Aldrich by Merck, Darmstadt, Germany), and absolute ethanol (Sigma-Aldrich by Merck, Darmstadt, Germany).

### 2.2. Methods

#### 2.2.1. Nanoparticles Manufacturing

The hot microemulsion technique was used to manufacture the solid lipid nanoparticles.

After a detailed study, a stable and suitable formulation was developed [10].

The lipid matrix for SLN-1 was composed of vegetable grade 50 stearic acid, representing 39.5% of the formulation; and for SLN-2, the lipid matrix was composed of vegetable grade 50 stearic acid and cholesteryl oleate (1:1.5), representing 39.5% of the formulation. For both formulations, octadecylamine was the cationic lipid used as the charged carrier, accounting for 47.5% of the formulation. Chondroitin sulfate sodium (CHON), representing 5% of the mixture, and Poloxamer, 188 corresponding to 8% of the formulation, were contained in the aqueous matrix.

The first step was that the raw materials were weighed individually. The aqueous solution of CHON in ultrapure water was prepared.

The lipid phase components and the aqueous phase components were heated separately to a temperature above their melting point (80 °C). They were then mixed and stirred at 80 °C at a speed of 20,000 rpm for 10 min to obtain a hot, turbid emulsion.

The emulsion was then immediately dispersed into refrigerated ultrapure water at 4 °C to produce the core solidification of the CHON-SLN. Next, the emulsion was centrifuged at defined stirring rates (19,000 rpm) and reaction times (every 15 min). The emulsion was then filtered through two qualitative filter papers of 43–48 μm and 7–9 μm and dosed into 5 mL glass vials hermetically sealed with an elastomeric stopper and stored.

Starting from the best-defined formulation conditions, other nanoparticles were manufactured, introducing changes in the amount or composition of the ingredients, as shown in Table 1.

In order to improve the stability of the formulations, lyophilization was decided upon for the testing of different cryoprotectants. Concentrations of 5 and 10% of trehalose, mannitol, povidone K30, and glycine were tested. The formulations were mixed via gentle magnetic stirring with an aqueous solution of the cryoprotectant to be tested. The lyophilization process was carried out with the Lyobeta 20 pilot freeze-drying system (Telstar, Terrassa, Spain). The same lyophilization cycle was used for all the formulations. First, samples were frozen at −45 °C for 4 h, and they were then lyophilized at a condenser temperature of −70 °C with vacuum pressure of 0.005 mbar for a period of 48 h. Tests for the selection of the best cryoprotectant were carried out with the SLN1 formulation.

#### 2.2.2. Characterization of SLN Formulations

- Morphological analysis by transmission electron microscopy (TEM)

The morphology of the samples was evaluated using a Tecnai Spirit microscope equipped with a LaB6 cathode (FEI Company, Hillsboro, OR, USA). Images were recorded at 120 kV using a 1376 × 1024-pixel CCD Megaview camera. The samples were negatively stained with a 2.0% uranyl acetate solution and absorbed onto carbon-coated copper grids CF200-Cu.

The samples were applied to 200 mesh formvar-carbon TEM copper grids by directly floating the grids on 40 µL drops of the sample, without any post-staining. The grids were washed twice with freshly filtered MQH_2_O to remove any excess sample. The stained grids were then allowed to dry overnight before imaging with JEOL1010 TEM at an accelerating voltage of 80 kV.

- Morphological analysis by atomic force microscopy (AFM)

Nanoscope Analysis (Bruker) software v1.40r1 was used for particle detection and analysis. Images were taken at 1 Hz and 512 × 512 pixels.

The preparation of the sample for the morphological analysis of the different isolated SLN formulations was carried out as follows: 20 µL of the diluted SLN sample (100 µL of the nanoparticles in 1 mL of MQH_2_O) were deposited onto a flat glass slide, previously glued on a Teflon disc with a two-component epoxy. It was left to rest for 2 min, and the sample was then gently rinsed with MQH_2_O for the photographs to be taken later in liquid. Before starting the imaging process, the samples were allowed to stabilize for 2 min to thermally equilibrate the sample and the AFM probe with the environment. This thermal equilibration is undertaken to avoid the piezo scanner drift.

- Determination of surface charge (zeta-potential) and particle size

In the process of SLN formula optimization, analyses of SLN sizes were determined via laser diffraction on a Mastersizer 2000 (Malvern Instruments, Malvern, UK) equipped with a 4 mW He–Ne laser (633 nm). For the treatment of samples in solution, the stirring speed was fixed at 800 rpm. The results are expressed as average surface diameter (D [3.2]) in nanometres (nm), unless otherwise indicated. The zeta potential of some formulations was also measured by laser Doppler microelectrophoresis in a Zetasizer Nano-Z (Malvern Instruments, UK). The zeta-potential values were obtained from the electrophoretic mobility of the nanoparticles and lipoplexes under an electric field. All samples were analyzed in triplicate and the results are expressed as mean voltage, in millivolts (mV) or nanometres (nm), depending on the measurement.

- Differential scanning calorimetry (DSC)

Differential scanning calorimetry was used for the evaluation of possible interactions between formulation components by means of a Mettler-Toledo DSC-822e calorimeter (Mettler-Toledo, Barcelona, Spain). Experimental conditions: aluminium crucibles of 40 μL volume, atmosphere of dry nitrogen with 50 mL/min flow rate, and heating rates of 10 °C/min. The calorimeter was calibrated with indium of 99.99% purity (m.p.: 156.8 °C, ΔH: 28.59 J/g).

Excipients alone, CHON, and mixtures of the excipients and CHON together were analyzed. They were weighed into a 40 μL aluminium pan, and an empty pan was used as a reference. Dry nitrogen (50 mL/min) was used to perform the assay in a nonoxidative atmosphere.

- X-Ray Diffraction

X Ray powder diffraction analysis was used to analyze the degree of crystallinity and the identity of the crystalline components of the nanoparticles. A PANalyticalX’Pert PRO MPD Ɵ/Ɵ solid sample diffractometer (Malvern Instruments, Malvern, UK) was used to obtain an X-ray diffraction pattern of the bulk components and a molten mixture of CHON and octadecylamine, with a radius of 240 mm in a configuration with converging beams that give rise to monochromatic X-rays. This impinges on a focusing mirror and forms a transmission geometry with the planar samples sandwiched between low absorption films. The pulverized samples studied were sandwiched between 3.6-micron thick polyester films. Experimental conditions were set as follows: Cu K α radiation (λ = 1.5418 Å) and working power of 45 kV–40 mA. The active length (aperture) of the PIXcel detector was 3.347°. Two Ɵ/Ɵ sweeps from 2 to 60° were performed with a step size of 0.026° and a measurement time of 200 s per step. The incident beam slits define a beam height of 0.4 mm, with 0.04 Söller radians, in both the incident and diffracted beams [30].

- Entrapment efficiency of CHON (EE%)

The determination of the EE% was carried out via two different validated methods: the titration method and the UV-VIS spectroscopy method.

  - Titration method:

Having considered the CHON determination assay described in the European Pharmacopeia and the American Pharmacopeia [31,32], this methodology was proposed to carry out a visual titration of CHON by means of a precipitation reaction.

A linear regression curve was prepared, with different concentrations of CHON reference solution in MQH_2_O (from 0.10 mg/mL to 1.20 mg/mL). A total of 15.0 mL of each—reference solution, samples of SLN containing CHON or plain SLN—were titrated. 1-cetylpyridinium chloride monohydrate (1.0 mg/mL) was used as the titrant. The solution turned turbid. At the end point, the liquid appears clear, with an almost-white precipitate in suspension. A total of 0.1 mL of a 1% solution of methylene blue R was added before starting the titration.

From the regression equation, using the volumes of the titrant consumed as a function of the concentrations of the CHON standard solutions, the concentration of free CHON in the sample was determined and calculated with the value of r^2^.

y = m × x + b(1)

with y denoting the mL titrant, and x denoting the mg/mL free CHON.

Knowing the initial concentration of the CHON used per sample, the CHON difference that constitutes the CHON bound to the nanoparticles was calculated. The entrapment efficiency of CHON (EE%) was calculated as well.

EE% = (Cx)/C × 100(2)

where C denotes mg/mL initial CHON, and x denotes the mg/mL free CHON in the analyzed sample.

The matrix was titrated for each type of SLN formulation.

Three different techniques were used to extract chondroitin sulfate from the nanoparticle: acid treatment, heat break, and alkali treatment. Alkali treatment was the only technique that allowed chondroitin sulfate to be extracted.

In the alkaline treatment, 10 mL of 0.7% *w/v* NaOH was added in accordance with the stoichiometric ratio, with the stearic acid present in the formulation. It was then stirred gently and left to react for 5 min at room temperature. Finally, the sample was analyzed using the titration method. Each sample was analyzed in triplicate.

  - UV-VIS spectroscopy method:

The CHON concentration in the nanoparticle samples was determined using a modified method [33,34], which consisted of the acid hydrolysis of glycosaminoglycans (CHON hydrolysis in monosaccharides) [35] and the reaction of hexuronic acid with carbazole. This obtained a red solution, colorimetrically quantifiable.

A total of 1 mL of the sample to be analyzed was added (a) either as a standard solution of glucuronic acid (100, 50, 25, and 10 mg/L) to a previously prepared solution of sodium tetraborate decahydrate, or (b) in sulfuric acid (0.08 mg/mL) in an ice-cold stoppered test tube. It was stirred and heated at 95–100 °C for 30 min. Then, it was cooled in an ice bath, and 0.20 mL of carbazole solution, prepared at a concentration of 1.25 mg/mL of absolute ethanol, was added. After this, it was shaken vigorously and heated for another 30 min.

It was then cooled to room temperature, and the final absorbance (A) of the standard solution or of the samples under study were measured at 520 nm and compared against a blank prepared in the same way. The value of the corresponding blank was subtracted from each absorbance value. MQH_2_O was used for standard solutions, and their respective plain SLN formulations were used for SLN. The analysis of each sample was carried out in triplicate. The linear regression equation of the absorbance of the standard solutions (Ast) against their concentration (Cst mg/L) was calculated. It was calculated with the value of r^2^, and the hexuronic acid content was calculated from the calibrator function, expressed as glucuronic acid in each sample. A conversion factor of 0.343 was used to convert the mg of the glucuronic acid found to mg CHON of bovine origin.

% CHON = (Cmg/mL)/(initial CHONmg/mL × 0.343) × 100(3)

#### 2.2.3. Stability Studies

The stability of the optimized SLN formulations was evaluated using climatic chambers (Heraeus, Hanau, Germany) and a refrigerator (LG, Seoul, Republic of Korea).

The stability studies were undertaken at 4 °C, 25 °C/60% RH, and 40 °C/75% RH for aqueous nanoparticles and 25 °C/60% RH for freeze-dried formulations.

The fresh samples (aqueous nanoparticle suspension) of the formulations were stored in hermetically sealed 5 mL glass vials with an elastomeric stopper, and the freeze-dried samples were kept in 5 mL hermetically sealed glass vials with an elastomeric stopper and an aluminium cap.

The stability was assessed at different points between 0 and 28 days of age (both inclusive) for fresh samples and 0, 4, 6, 8, and 11 months for lyophilized samples following storage.

At each pull time, the formulations were tested and not returned to the stability chamber. The formulations were assessed in terms of the parameters considered benchmarks for establishing the stability of SLN, including the determination of the surface charge (zeta-potential), the particle size, and the entrapment efficiency of the CHON (EE%).

## 3. Results and Discussion

### 3.1. Characterization of SLN Formulations

- Morphological analysis by transmission electron microscopy (TEM)

**Figure 1:**
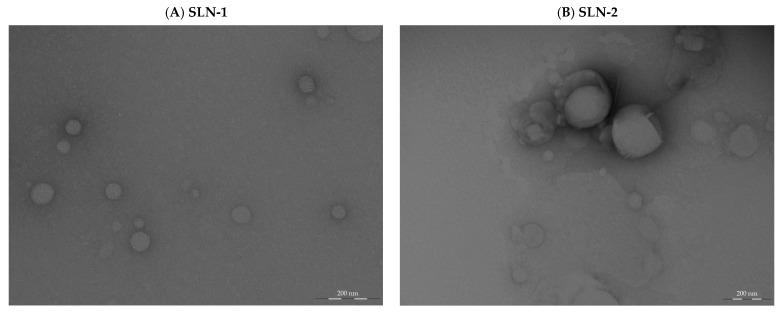
TEM images of nanoparticles containing CHON in aqueous medium (scale bar = 200 nm): (**A**) TEM image of SLN-1 nanoparticles; (**B**) TEM image of SLN-2 nanoparticles.

The morphology of the nanoparticles in the original aqueous dispersion was investigated using TEM, and the data generated are depicted in Figure 1.

The images reveal that circular nanoparticles with various sizes could be seen, and that they were smaller in size, with an average diameter less than 200 nm. Studies indicate that the size range of 20–300 nm enhances drug delivery efficiency, particularly for transdermal applications. This size range is effective because smaller particles have the ability to disrupt the lipid structure of the stratum corneum, the outermost layer of the skin. This approach enables nanoparticles to induce transient disruptions or openings in the skin’s protective barrier, thereby enhancing the permeation of therapeutic agents across the epidermis and dermis layers. This mechanism improves the absorption and effectiveness of the delivered drugs, allowing for enhanced therapeutic outcomes [36,37].

- Morphological analysis by atomic force microscopy (AFM)

The AFM technique was used to characterize and confirm the morphology of the nanoparticles under study. This analysis allowed us not only to observe the image in two dimensions but also to profile the height and diameter of the particles, as well as to see the morphology in three dimensions.

**Figure 2:**
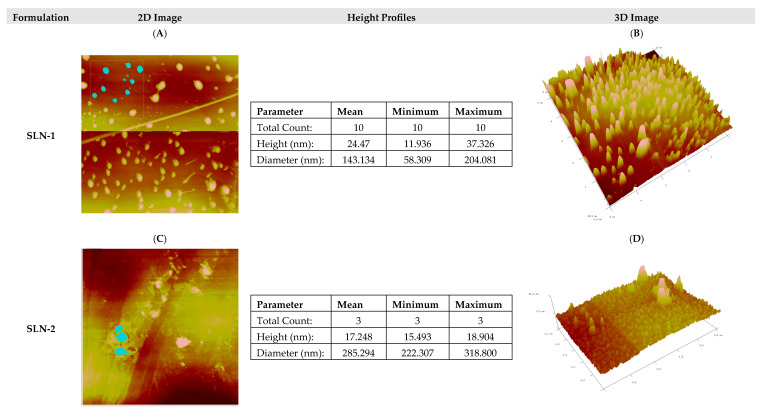
Typical AFM measurement of fresh nanoparticles. The nanoparticles marked in turquoise are those used in the height profiles: (**A**) 2D image of SLN-1 nanoparticles; (**B**) 3D image of SLN-1 nanoparticles; (**C**) 2D image of SLN-2 nanoparticles; (**D**) 3D image of SLN-2 nanoparticles.

As can be seen in Figure 2, the height profile and morphological visualization by AFM were carried out on the formulations. The nanoparticles were evaluated freshly manufactured and in an aqueous medium. The first column corresponds to the two-dimensional images, and the particles in turquoise color correspond to the nanoparticles used to create a height and size profile per formulation.

The nanoparticles remain in the range of less than 200 nm in diameter, except for SLN-2, whose morphology showed a mean size of 285.3 nm. In all cases, nanoparticles with heterogeneous sizes and varied shapes were observed, with a predominance of circular shapes. 

**Figure 3:**
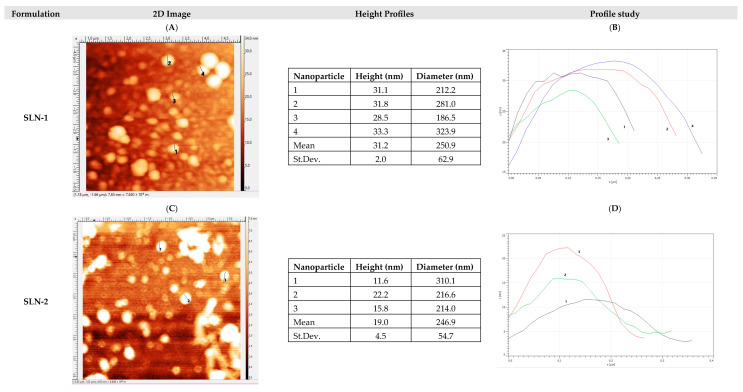
Stability study of the SLN after 8 months of lyophilization. The formulations were lyophilized and stored at 25 °C/65% HR. Visualization was conducted via typical AFM nanoparticle (SLN + CHON) measurement: (**A**) 2D image of SLN-1; (**B**) profile study of SLN-1; (**C**) 2D image of SLN-2; (**D**) profile study of SLN-2.

Figure 3 corresponds to the morphological study by AFM after 8 months of storage at 25 °C/60%HR and in a lyophilized form of the SLN-1 and SLN-2 formulations. In all cases, the nanoparticles were found to be heterogeneous in size and circular in shape, with an average diameter ranging from 200 nm to 250 nm. The heights remained similar to those found when manufacturing the nanoparticles and studying them in an aqueous medium (Figure 2).

Fresh SLN-1 presented a height range between 11.9 and 37.3 nm, while the lyophilized samples, which were held stable for 8 months at 25 °C/60%RH, showed a range between 28.5 and 33.3 nm. In the case of fresh SLN-2, it presented a height ranging from 15.4 to 18.9 nm, while for the lyophilized samples, after being held stable for 8 months at 25 °C/60%RH, they showed a range between 11.6 and 22.2 nm.

Therefore, it was demonstrated that nanoparticles are morphologically stable for at least 8 months after manufacturing and lyophilization.

- Determination of surface charge (zeta-potential) and particle size

The different formulations, recently formulated and in an aqueous medium, were characterized by their size and surface charge. Table 2 shows the particle sizes reported as D_50_, and the zeta potential, with both characteristics measured in triplicate. The sizes are generally around 121 nm, and the zeta potential is around −40 mV.

The zeta potential is considered a primary indicator of the stability of the dispersion of nanomaterials, and by convention, a value below −25 mV and above 25 mV is considered stable. Among the factors that affect the obtained zeta potential value, the concentration and type of ions, as well as the pH of the solution, strongly affect the zeta potential [38].

The mean values are around −40 mV, which are considered stable and the most suitable values for the nanoparticle preparations studied.

- Differential scanning calorimetry (DSC)

The DSC thermograms were analyzed with the Mettler Toledo Star System program, recording the onset of each relevant peak and quantifying the enthalpy values corresponding to each endotherm. The potential interactions were hypothesized from the variations of these parameters in the thermograms of the mixtures with respect to those observed in the thermograms of the pure components [39,40,41].

The formulation was characterized by DSC in order to assess a potential incompatibility between excipients and CHON in terms of thermal stability. The DSC technique can be used in the study of the interactions between drugs and excipients and, therefore, also in the study of their compatibility [42,43,44]. In general, the interpretation is based on the modifications observed in a DSC analysis of the drug in the presence and absence of the excipient.

Although it is accepted that a change observed in a DSC analysis of the active ingredient–excipient mixture is irrefutable evidence of an interaction different to that in the case of the pure drug, it does not necessarily imply incompatibility.

The changes observed in the thermal properties of the mixtures are due to an interaction between drug and excipient induced by heating, but it is possible that at room temperature, this interaction is negligible. Likewise, it is likely that a physicochemical transformation of one species (change of state) will alter the environment of the other and cause it to have a different thermal behavior. It is also possible that the interaction between an active ingredient and an excipient is very slow at room temperature but, at a higher temperature, has appreciable kinetics and therefore results in a modification to its DSC thermogram. In short, the DSC technique provides useful information regarding the interaction/compatibility between an active ingredient and an excipient in the following cases:

1. If the thermogram of the mixture is a superposition of the individual thermograms, then there is no interaction, and the species are considered compatible.
2. If a new thermal phenomenon appears at a lower temperature than the first thermal phenomenon observed for any of the species separately, then interaction has taken place, and the species are considered incompatible.

**Figure 4:**
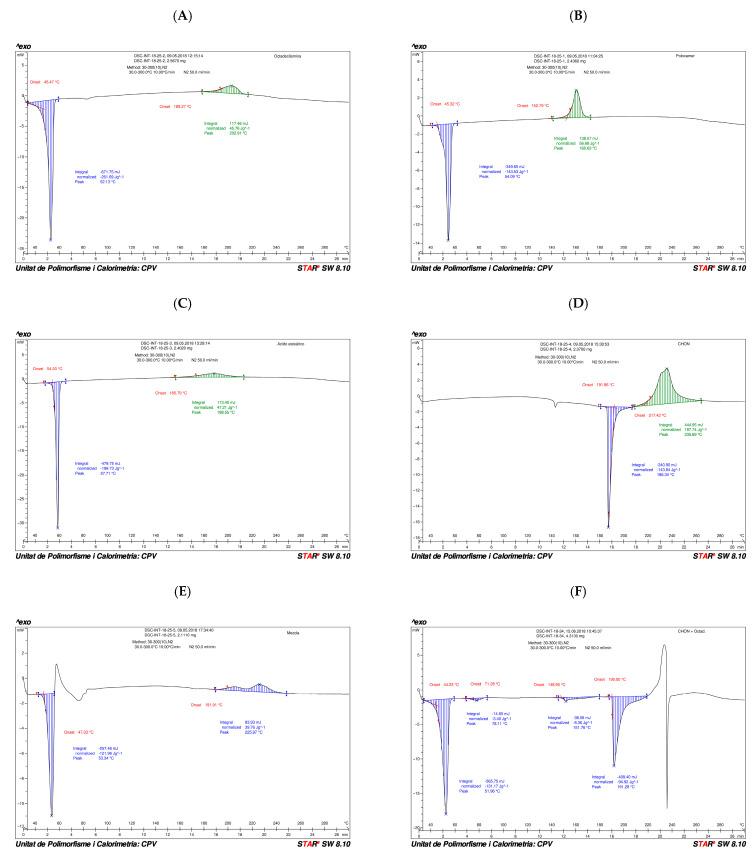
DSC thermograms obtained in the study of potential CHON–excipient interactions: (**A**) octadecylamine; (**B**) poloxamer; (**C**) stearic acid; (**D**) CHON; (**E**) mixture of the components of the SLN formulation in the original proportion; (**F**) mixture of CHON and octadecylamine in the original proportion of the SLN formulation.

Figure 4 shows the DSC thermograms of CHON and of each of the excipients in pure form, all of them presenting defined endotherms, corresponding to melting or melting/decomposition processes. In addition, the DSC thermograms of the CHON mixture with the three excipients of the formulation and the 1:1 mixture of the main excipient, octadecylamine, are shown.

- The DSC thermogram of octadecylamine (Figure 4A) presents an initial endothermic phenomenon starting at 45 °C, with an associated heat of 261.69 J/g, and an exothermic phenomenon starting at 189 °C, with an associated heat of 45.76 J/g, corresponding to the degradation of octadecylamine when subjected to a high temperature.
- The DSC thermogram of poloxamer (Figure 4B) shows an initial endothermic event starting at 45 °C, with an associated heat of 143.53 J/g, and an exothermic event starting at 152 °C, with an associated heat of 56.68 J/g, corresponding to the degradation of poloxamer when it is subjected to a high temperature.
- The DSC thermogram of stearic acid (Figure 4C) shows an initial endothermic event starting at 54 °C, with an associated heat of 199.73 J/g, and an exothermic event starting at 165 °C, with an associated heat of 47.21 J/g, corresponding to the degradation of stearic acid when it is subjected to a high temperature.
- The DSC thermogram of CHON (Figure 4D) shows an initial endothermic event starting at 191 °C, with an associated heat of 143.84 J/g, and an exothermic event starting at 217 °C, with an associated heat of 187.74 J/g, corresponding to the degradation of CHON when it is subjected to a high temperature.
- The DSC thermogram of the mixture of the four substances in the same proportions as required in the manufacture of SLN (Figure 4E) shows an initial endothermic phenomenon starting at 47 °C, with an associated heat of 121.46 J/g, followed by an exothermic event (not evaluated since it is concomitant to the previous endothermic event) and an exothermic phenomenon starting at 191 °C, with an associated heat of 39.76 J/g. Since the percentage of CHON in the formulation mixture is only 5%, its melting phenomenon has not been observed, which, in addition, is also obscured by the superposition of the decomposition of the excipients in the heated mixture. Moreover, the exothermic event, which occurs concomitantly to the melting of the lowest melting excipient (the octadecylamine), can be attributed to a recrystallization event from the partially liquid mixture at around 50 °C. This phenomenon is followed by a broad endothermic event, probably as a consequence of the melting of the remaining solid excipients. Thus, according to the general rules previously described concerning the compatibility of excipients, we cannot discard a potential interaction between excipients upon heating. However, the low proportion of the active compound in the mixture precludes of a definitive conclusion.
- For this reason, the possible interaction between CHON and the main excipient of the mixture, octadecylamine, was further studied (Figure 4F). The thermogram of the binary mixture, CHON–octadecylamine, shows an initial endothermic phenomenon from 44 °C, with an associated heat of 131.17 J/g; a second endothermic phenomenon from 71 °C, with an associated heat of 3.40 J/g; a third endothermic event starting at 148 °C, with an associated heat of 8.36 J/g; and a fourth endothermic event starting at 190 °C, with an associated heat of 94.92 J/g. Interestingly, the main melting events of both compounds appear essentially at the same temperature onsets. Moreover, secondary endothermic thermal events, which we have not studied in detail, can still be observed. Only the decomposition of the mixture is mostly altered. For all the foregoing, it is considered that the interaction is likely to be negligible.

In summary, the DSC experiments suggest that although excipients’ interactions have been observed, this does not seem to alter the stability of the active compound, CHON, in the mixture. However, alternative stability experiments should be conducted in the future to further clarify this issue.

- X-Ray Diffraction

**Figure 5:**
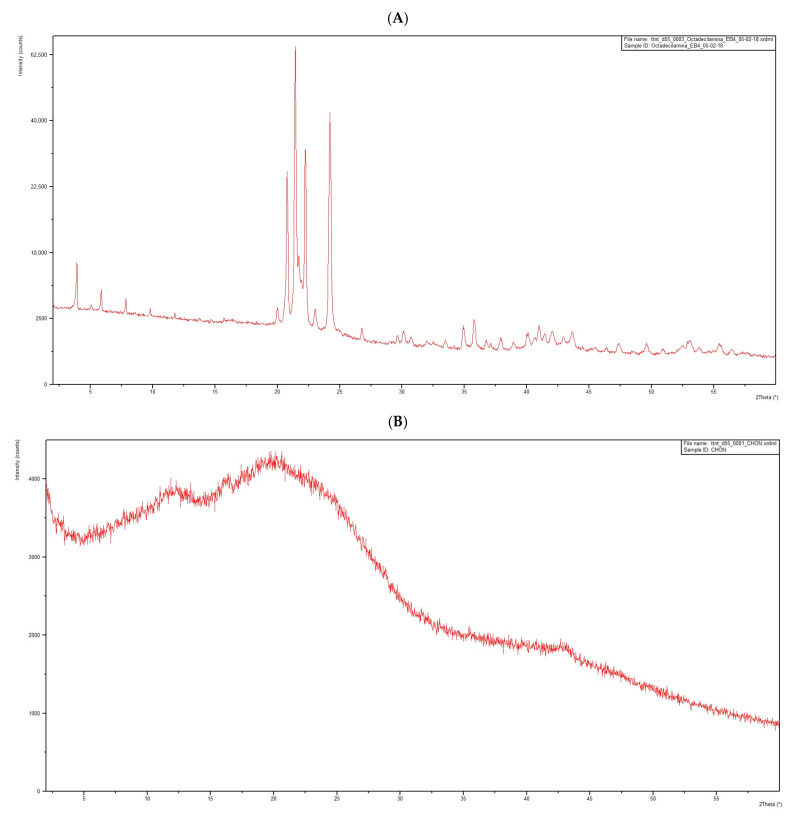

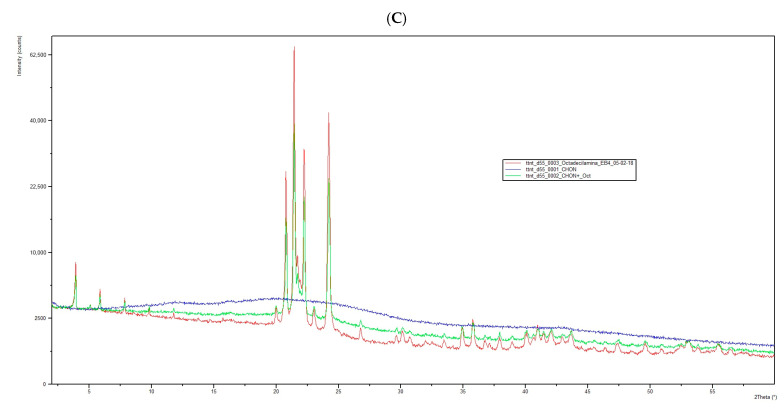
Powder X-ray diffraction patterns of octadecylamine (**A**), CHON (**B**), and a mixture (1:1) of CHON and octadecylamine (**C**).

Powder X-ray diffraction was also used to support the observation that the main excipient does not interact in the solid state at room temperature. To back up this observation, the PXRD pattern of the individual compounds and the 1:1 physical mixture were measured. Figure 5A,B show the patterns of the pure octadecylamine and CHON, respectively. It can be seen that the excipient is essentially crystalline, while the drug compound displays the characteristic halo of an amorphous phase. On the other hand, Figure 5C shows that the physical mixture can be considered a perfect superposition of both diffraction parameters, revealing that neither of the new solid phases have been formed during the preparation of the mixture. Furthermore, no change has occurred in the crystallinity of the excipient nor in the drug compound. This is additional evidence to reinforce the hypothesis that CHON can be considered stable in the presence of the excipients used for the formulation of chondroitin sulfate-loaded solid lipid nanoparticles.

- Entrapment efficiency of CHON (EE%)
  - Titration method:

The volumetric titration method was first validated and then used in the initial studies to determine the encapsulation efficiency of CHON (EE%). The data validation method is shown under the Supplementary Materials section [45,46,47,48].

In order to extract the chondroitin sulfate, an excess of NaOH was added in accordance with the stoichiometric ratio, with the stearic acid present in the formulation. An irreversible saponification reaction took place, releasing the encapsulated CHON, nd it was also noted that the base used did not interfere in the reaction with the titrant.

As can be seen in Table 3, in the SLN samples containing CHON and previously treated with NaOH, when evaluated by volumetric titration, 93% of free CHON was found. NaOH forms an irreversible saponification reaction with stearic acid, which breaks down the nanoparticle and releases CHON. When sodium hydroxide and stearic acid react with each other, this results in the formation of sodium salt and water.

These tests show that the CHON remained bound to SLN during titration, and that only the CHON not bound to nanoparticles reacted. Therefore, this method was discarded for use in the determination of CHON’s encapsulation efficiency (EE%) due to its low sensitivity.

  - UV-VIS spectroscopy method:

The UV-VIS spectroscopy method was validated and used in subsequent studies to determine the encapsulation efficiency of CHON (EE%). The data validation method is shown in the Supplementary Materials section.

As can be seen in Table 4, the values obtained are similar in both formulations and higher than 75% in all the samples analyzed, reaching values close to 100% in the case of SLN-2.

The UV-Vis method allows smaller sample volumes to be analyzed and obtains better results than the titration method.

### 3.2. Nanoparticles Manufacturing: Lyophilization Process

The best cryoprotectant was chosen based on four results: particle size, surface charge (z-potential), cake, and the reconstituted appearance. All the vials were re-dispersed in ultrapure water (3.0 mL).

The results of eight formulations are shown in Table 5.

**Figure 6:**
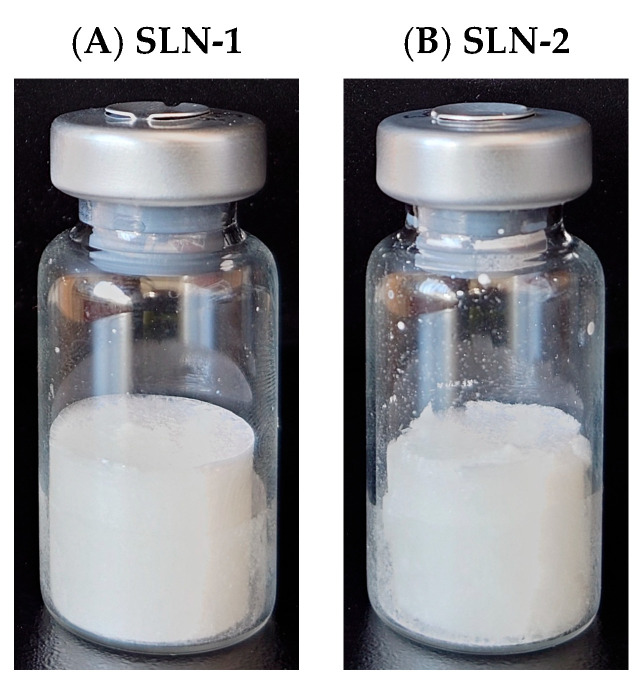
SLN-1 and SLN-2 lyophilizate samples with 5% of Trehalose as cryoprotectant: (**A**) image of SLN-1 lyophilizate nanoparticles; (**B**) image of SLN-2 lyophilizate nanoparticles.

After the evaluation of the results, the best cryoprotectant was shown to be trehalose, regardless of the supplier and the concentrations studied. Figure 6 shows the appearance of the lyophilized vials, with 5% trehalose as cryoprotectant. The cake obtained is correct, confirming the suitability of the freeze-drying cycle used.

Batches were produced using Trehalose Panreac^®^ at a concentration of 5%, selected as the minimal effective dose of cryoprotectant. These findings are in line with the results reported in previous studies conducted by other researchers [49,50,51].

### 3.3. Stability Studies

To establish the stability of the nanoparticles, the surface charge (zeta potential), particle size, and encapsulation efficiency (%) were determined.

The stability of fresh nanoparticles was evaluated at different temperatures: 4 °C, 25 °C/60% RH, and 40 °C/75% RH. The evaluated nanoparticles were SLN-1 and SLN-2.

As observed in the results set out in Table 6, for the initial formulation, as revealed by Fábregas [30,52], the formulation shows a progressive increase in nanoparticle size at 4 °C and 25 °C/60% RH but remains stable at 40 °C/75% RH. At 7 days of stability, at 4 °C, the nanoparticle size value is 616 nm, and at 25 °C/65% RH, the nanoparticle size value is 43,000 nm. The zeta potential is maintained in the tens around −40 mV.

The encapsulation efficiency is correct as it remains above 75%. When the particle size increases considerably, as observed in the SLN-1 formulation, we decided not to perform the test because the encapsulation is considered inadequate since this increase in size could be due to the formation of particle agglomerations, which would not be able to release the encapsulated active ingredient effectively.

It can be concluded that the SLN-1 formulation is stable in an aqueous medium for 9 days at 40 °C/75% RH.

As commented above, in the SLN-1 formulation, it was observed that the particle size increases at room temperature and under refrigeration, but not at 40 °C. This phenomenon can be attributed to the effect of temperature on particle stability and the propensity for aggregation. At lower temperatures, such as those in the refrigerator, or at room temperature, reduced thermal energy limits molecular movement and the rearrangement of stabilizers. This can promote aggregation or coalescence, resulting in an increase in particle size. In contrast, at 40 °C, the higher thermal energy helps to stabilize the lipid layers, preventing particles from melting and preserving size stability, even under short-term stress. These findings have been corroborated by other researchers [22,53,54].

In the case of the SLN-2 formulations, the stability data show the presence of nanoparticles at the three temperatures evaluated up to 28 days after their manufacture, since cholesteryl oleate gives greater stability to the formulation over time. The surface charge ranges from −25.8 to −51.6 mV, apparently not affecting the presence of nanoparticles smaller than 200 nm. The encapsulation efficiency is correct during the 28 days in all stability conditions.

The aqueous formulation SLN-2 shows a good stability at the three temperatures evaluated at 28 days, maintaining similar values in terms of particle size, zeta potential, and encapsulation efficiency throughout the stability test.

Table 7 lists the values obtained at different time intervals, depending on the nanoparticle studied. Formulations SLN-1 and SLN-2 present average particle sizes between 114 and 400 nm, measuring their values up to 6 and 8 months after their being produced.

SLN-1 and SLN-2 present particle sizes around 400 nm after 8 months of lyophilization. However, this variation, with respect to previous values, may be due to the equipment used, or to the fact that the particles were not completely resuspended, and the necessary time was not allowed to go by for the separation of the nanoparticles in the aqueous suspension.

Nanoparticle tracking analysis (NTA) technology was used for the analysis of some samples. This technique measures the concentration of particles per millilitre of suspension. Formulations SLN-1 and SLN-2 were analyzed with this technique at 4 and 11 months.

If the particle size values obtained with the Mastersizer 2000 (Malvern Instruments, Malvern, UK) (Table 7) are compared with those obtained with the NTA (Table 8), in both cases, an increase in particle size with storage time is observed. However, it should be noted that using NTA, the mean sizes in all cases remain, showing only very small variations from one measurement to another. The nanoparticles show mean sizes in the range of 149.2 to 276.4 nm. The concentration of the nanoparticles is to the 10^11 power, except for the SLN-1 formulations, in which a slight decrease in the number of nanoparticles over time and an increase in their size due to nanoparticle aggregation processes were observed.

Stability studies of the lyophilized SLN-1 and SLN-2 formulations show they have good stability at 25 °C/60% RH temperatures evaluated over 8 months, maintaining similar values of particle size, zeta potential, and encapsulation efficiency throughout the stability test.

## 4. Conclusions

This research successfully developed and characterized chondroitin sulfate-loaded solid lipid nanoparticles (CHON-SLNs) using a hot microemulsion technique. This study highlights several key findings that draw attention to the potential of these nanoparticles for therapeutic use.

The hot microemulsion technique, combined with lyophilization using trehalose as a cryoprotectant, allowed the production of stable SLN formulations with high encapsulation efficiency, particularly for SLN-2. This formulation neared 100% encapsulation efficiency, demonstrating its efficacy in retaining chondroitin sulfate within the nanoparticles. Trehalose emerged as the optimal cryoprotectant, effectively preserving the structural integrity, functionality, and reconstitution quality of the nanoparticles, even after extended storage periods.

Analytical techniques, including TEM, AFM, DSC, and XRD, confirmed the uniform morphology and compatibility of the nanoparticle components, minimizing degradation risks. TEM and AFM analyses revealed that the nanoparticles retained a stable, circular structure with a particle size under 200 nm, even after six months. Zeta potential measurements around −40 mV further supported strong colloidal stability under varying environmental conditions, including temperatures of 4 °C, 25 °C/60% RH, and 40 °C/75% RH. The differential scanning calorimetry (DSC) results indicated no significant chemical interactions between SLN components under standard storage conditions, though slight interactions between chondroitin sulfate and octadecylamine were observed at elevated temperatures.

Lyophilization significantly enhanced the stability of CHON-SLNs, preventing high aggregation and degradation over time. The freeze-dried nanoparticles retained their particle size and zeta potential for up to eight months under controlled conditions. The stability and scalability of this approach position these formulations as robust options for drug delivery systems aimed at targeted and sustained-release therapies.

SLN-2 demonstrated superior stability over time and under varying environmental conditions, making it the most viable formulation for long-term applications. The combination of chondroitin sulfate’s regenerative properties and the biocompatibility of SLNs positions this formulation as an effective option for localized therapeutic delivery, particularly for treating inflammatory and degenerative diseases.

In conclusion, the CHON-SLN formulations represent a versatile, customizable, and scalable strategy for enhancing drug stability and therapeutic efficacy. The adaptability of modifying the lipid matrix composition and cryoprotectant concentration expands their potential applications. Future research should conduct in vivo studies to further validate the clinical applicability of these systems and tailor their use to various therapeutic needs.

## Figures and Tables

**Table 1:** Composition of the solid lipid nanoparticles studied.

Composition (%)	Formulation	
	SLN-1	SLN-2
CHON	5	5
Stearic acid	40	16
Cholesteryl oleate	-	24
Octadecylamine	48	48
Poloxamer 188	8	8
**Total (%)**	**100**	**100**

**Table 2:** Average particle size (D_50_) and zeta potential of SLN with CHON in different formulations.

Formulation	Particle Size (nm)	Zeta Potential (mV)
SLN-1	121 ± 8	−41.8 ± 4.3
SLN-2	121 ± 6	−41.9 ± 2.5

**Table 3:** Measurement of the percentage of free CHON found in the samples analyzed via the titration method. SLN-CHON + NaOH: the same samples previously treated with NaOH (0.7% *w*/*v*).

Sample	SLN-CHON + NaOH (%)
1	93.22
2	97.53
3	94.93
4	97.37
5	86.17
Mean ± SEM	93.84 ± 2.079 N = 5

**Table 4:** CHON encapsulation efficiency in different SLN formulations via the spectrophotometric method.

Sample	SLN-1	SLN-2
1	77.6	88.1
2	76.4	86.2
3	78.8	81.4
4	83.4	82.3
5	80.2	87.0
6	82.5	87.2
7	74.9	84.8
8	83.7	82.1
9	83.7	98.3
10	83.1	81.6
11	-	91.5
12	-	93.6
13	-	89.1
Mean (%)	80.4	87.2
Standard deviation	3.3	5.1
Standard error of the mean	1.1	1.4

**Table 5:** Results of the characterization of the eight cryoprotectant-based formulations (from SLN1 formulation).

Cryoprotectants	Concentration	D (0.5) (nm)	Z-Potential (mV)	Macroscopic Aspect	Reconstitution
Povidone K30	5%	668.474	−5.17	Collapsed yellowish	No aggregates; long reconstitution time
		251.000	−4.63		
		202.000	−5.42		
Povidone K30	10%	1005.841	−6.51	Collapsed yellowish	No aggregates; long reconstitution time
		225.000	−6.23		
		175.000	−6.85		
Glycine	5%	97.694	−45.0	Correct and compact; the cake broke over time	Microscopic aggregates
		103.884	−51.8		
		116.957	−46.3		
Glycine	10%	104.384	−56.4	Correct and compact; the cake broke over time	Microscopic aggregates
		115.373	−51.6		
		142.348	−57.8		
Glycine	5%	72.297	−2.85	Correct and compact; the cake broke over time	Phase separation and aggregates
NaH_2_PO_3_	3.12%	50.871	−4.01		
Na_2_HPO_3_	3.56%	49.355	−4.16		
Glycine	10%	158.894	−1.71	Correct and compact; the cake broke over time.	Phase separation and aggregates
NaH_2_PO_3_	3.12%	50.667	−2.20		
Na_2_HPO_3_	3.56%	50.587	−3.06		
Mannitol	5%	123.497	−38.1	Unacceptable, brittle cake	Macroscopic aggregates
		115.142	−37.0		
		112.261	−37.3		
Mannitol	10%	127.939	−43.6	Unacceptable, brittle cake	Macroscopic aggregates
		107.501	−41.8		
		101.757	−46.6		
Trehalose Panreac^®^	5%	65.158	−47.3	Correct	No aggregates
		60.460	−48.4		
		58.672	−49.1		
Trehalose Panreac^®^	10%	68.672	−30.5	Correct	No aggregates
		61.978	−28.6		
		63.682	−31.3		
Trehalose Cerestar^®^	5%	59.561	−38.4	Correct	No aggregates
		63.408	−39.6		
		54.429	−42.8		
Trehalose Cerestar^®^	10%	84.113	−38.9	Correct	No aggregates
		69.195	−44.7		
		69.403	−44.6		

**Table 6:** Particle size (nm), zeta potential (Mv), and encapsulation efficiency (%) in the study of the stability of nanoparticles in an aqueous medium.

**SLN-1**																		
**Day**	**Temperature 4 °C**						**25 °C/60% RH**						**40 °C/75% RH**					
	Size (nm) D [3.2]	SD	Pot Z	SD	% EE	SD	Size (nm) D [3.2]	SD	Pot Z	SD	% EE	SD	Size (nm) D [3.2]	SD	Pot Z	SD	% EE	SD
0	**114**	1.1	**−40.0**	0.5	79.9	0.5	**114**	1.1	**−40.0**	0.5	**79.9**	0.5	**114**	1.1	**−40.0**	0.5	**79.9**	0.5
1	**138**	2.3	**−45.0**	0.3	79.8	0.4	**271**	5.0	**−45.3**	0.7	**79.7**	0.6	**123**	2.0	**−40.1**	0.3	79.8	0.6
2	**126**	4.4	**−46.2**	0.6	79.9	0.4	**274**	22.3	**−46.3**	0.2	**79.8**	0.4	**149**	6.1	**−40.3**	0.5	79.9	0.4
3	**163**	2.4	**−46.2**	0.8	79.8	0.6	**663**	32.6	**−47.6**	0.9	ND	ND	**191**	63.5	**−41.2**	0.4	79.9	0.5
6	**209**	6.1	**−49.1**	0.9	79.8	0.5	**39,000**	336.5	**−45.5**	0.2	ND	ND	**229**	58.7	**−42.8**	1.1	79.8	0.5
7	**616**	28.5	**−46.5**	0.5	ND	ND	**43,000**	3265.4	**−44.4**	0.3	ND	ND	**278**	31.1	**−44.8**	0.9	79.8	0.4
9 *	**1335**	216.6	**−45.9**	1.0	ND	ND	**47,000**	3413.8	**−45.6**	0.5	ND	ND	**336**	35.9	**−44.6**	0.6	79.8	0.6
**SLN-2**																		
**Day**	**Temperature 4 °C**						**25 °C/60% RH**						**40 °C/75% RH**					
	Size (nm) D [3.2]	SD	Pot Z	SD	% EE	SD	Size (nm) D [3.2]	SD	Pot Z	SD	% EE	SD	Size (nm) D [3.2]	SD	Pot Z	SD	% EE	SD
0	**124**	1.5	**−46.9**	0.5	**88.9**	0.5	**124**	1.5	**−46.9**	0.5	**88.9**	0.5	**124**	1.5	**−46.9**	0.5	**88.9**	0.5
3	**129**	2.9	**−50.0**	0.2	**88.7**	0.4	**118**	0.6	**−49.8**	0.5	**89.0**	0.5	**128**	12.7	**−51.3**	0.3	88.7	0.6
7	**129**	2.8	**−48.1**	1.4	**88.8**	0.5	**125**	3.2	**−51.6**	1.0	**88.9**	0.5	**125**	3.2	**−48.4**	0.2	88.8	0.6
14	**124**	2.6	**−50.8**	0.2	**88.7**	0.6	**127**	2.3	**−47.6**	1.7	**88.7**	0.6	**131**	10.4	**−40.4**	1.0	88.6	0.5
21	**143**	1.7	**−26.9**	0.4	**88.6**	0.6	**130**	3.1	**−47.6**	0.7	**88.8**	0.4	**123**	2.9	**−45.4**	1.0	88.7	0.5
23	**128**	2.0	**−31.4**	0.7	**88.7**	0.5	**130**	3.5	**−25.8**	0.9	**88.9**	0.4	**162**	21.2	**−48.5**	1.2	88.7	0.5
28	**213**	4.5	**−48.1**	0.8	**88.9**	0.5	**128**	1.7	**−42.4**	0.7	**88.8**	0.5	**139**	1.0	**−47.1**	1.2	88.8	0.4

Results marked in red indicate that the values obtained are extremely high and therefore incorrect. * It was decided to stop the stability study of this formulation due to the poor results obtained at 9 days or before. ND: not determined.

**Table 7:** Measurement of the particle size, zeta potential, and encapsulation efficiency of lyophilized nanoparticles after different storage times at 25 °C/60% RH.

SLN-1						
Date/Condition	Size (nm)	SD	Pot Z	SD	EE%	SD
Day 1_aquous medium	114	1.1	−40	0.5	79.9	0.5
Day 1_Freeze-dried	183	43.3	−39.3	1.2	79.8	0.6
Month 6_Freeze-dried	164	24.7	−43.8	1.7	79.7	0.4
Month 8_Freeze-dried	391.2	4.9	−32.55	1.08	79.6	0.4
**SLN-2**						
**Date/Condition**	**Size (nm)**	**SD**	**Pot Z**	**SD**	**EE%**	**SD**
Day 1_aquous medium	124	1.5	−46.9	0.5	88.9	0.5
Day 1_Freeze-dried	137	4.2	−33.2	0.6	88.7	0.5
Month 6_Freeze-dried	179	9.3	−25.8	0.7	88.7	0.5
Month 8_Freeze-dried	400.7	4.9	−22.76	1.29	88.6	0.4

**Table 8:** Measurement of the particle size of lyophilized nanoparticles after different storage times at 25 °C/60% RH and using nanoparticle tracking analysis software v1.40r1.

Sample	Time	Mean (nm)	D90 (nm)	Concentration (Particle/mL)
SLN-1	Month 4	149.2 ± 4.5	215.3 ± 12.8	1.55 × 10^11^ ± 1.40 × 10^10^
	Month 11	152.7 ± 2.0	267.1 ± 9.0	8.87 × 10^9^ ± 1.41 × 10^8^
SLN-2	Month 4	235.0 ± 3.4	319.9 ± 12.1	4.30 × 10^11^ ± 1.06 × 10^10^
	Month 11	276.4 ± 5.9	377.6 ± 18.2	1.59 × 10^11^ ± 7.18 × 10^9^

## Data Availability

The original contributions presented in this study are included in the article/Supplementary Materials. Further inquiries can be directed to the corresponding author.

## Supplementary Materials

The following supporting information can be downloaded at https://www.mdpi.com/article/10.3390/pharmaceutics17010086/s1, Table S1: Study of the selectivity of the Spectrophotometric Method, by determining the percentage of discrepancy in the signal obtained; Table S2: Linear regression results for the assay of linearity of CHON in SLN by titration method; Table S3: Linear regression results for the assay of linearity of CHON in SLN by UV-Vis spectrophotometric method: Table S4: Results of the accuracy determination for the study of CHON in SLN by titration method: Table S5: Results of the accuracy determination for the study of CHON in SLN by spectrophotometric method; Table S6: Results of intermediate precision determination for the study of CHON in SLN by titration method; Table S7: Results of intermediate precision determination for the study of CHON in SLN by spectrophotometric method; Table S8: Results of variation of the amount of volume of titrant in the determination of robustness for the study of CHON in SLN by titration method; Table S9: Results of Wavelength (nm) variation in the robustness determination for the study of CHON in SLN by spectrophotometric method; Table S10: Tukey’s Multiple Comparison Test for changes in wavelength in the robustness study; Table S11: Results of absorbance variation in the robustness determination for the study of CHON in SLN by spectrophotometric method; Table S12: Bonferroni’s Multiple Comparison Test for changes in the volume of sulfuric acid used in the robustness study; Table S13: Summary of the results obtained in the validation of the analytical methods for the determination of the EE% of CHON in the SLN; Figure S1: Linear regression curve for the assay of CHON in SLN; Figure S2: Linear regression curve for the assay of CHON in SLN.
