# Supplementary material for: Development and Characterization of Lyophilized Chondroitin Sulfate-Loaded Solid Lipid Nanoparticles: Encapsulation Efficiency and Stability
Source: Pharmaceutics. 2025 Jan 10;17(1):86. doi: 10.3390/pharmaceutics17010086 (PMC11768393; doi:10.3390/pharmaceutics17010086)
Supplement: Supplementary file 1 [file pharmaceutics-17-00086-s001.zip › pharmaceutics-3347567-supplementary.pdf]

pharmaceutics MDPI

Supplementary material 1 (S1)

Validation of analytical methods to determine EE%

1. Specificity

Specificity is a parameter to determine the ability of the method to distinguish between the analyte and the other substances or excipients present [1,2].

- ***Titration method***

Volumetric titration was performed in triplicate of the matrix that consisted of a formulation of nanoparticles without the presence of CHON and it did not show a consumption of titrating solution by the matrix. Therefore, no precipitate was formed, indicating that the method is specific to determine the presence of CHON and that the excipients of the formulation do not interfere in the analysis.

- ***UV-VIS spectrophotometric method***

In the case of the spectrophotometric method, the determinations made on the matrix of excipients show a signal from the matrix. For this reason, the effect of matrix interference on the determination of the active ingredient in the formulation was quantified.

For the selectivity of the spectrophotometric method, the percentage of discrepancy between the signal obtained by the CHON 0.4 mg/ml sample in aqueous solution was calculated with respect to a matrix sample enriched with CHON 0.4 mg/ml.

Table S1: Study of the selectivity of the Spectrophotometric Method, by determining the percentage of discrepancy in the signal obtained.

Determination	Matriz + CHON (Abs)	CHON in aqueous medium (Abs)
1	1.010	0.820
2	1.022	0.832
3	1.115	0.925
4	1.182	0.998
**Mean**	**1.082**	**0.894**
**SD**	0.081	0.084
**Discrepancy (%)**	**21.09**	
**Student´s t test**	**p < 0.0001**	

The t test, for the significance of differences among experimental groups ($p < 0.05$) was obtained by Student's t test, and it was found that there are significant differences, with a p value less than 0.0001.

In addition, as observed in **Table S1**, a positive bias of 21.09 % is observed, so it does not meet the selectivity criterion, since the value of the percentage of discrepancy is greater than 5 %.

This high discrepancy value is partly due to the varied and lipid nature of the matrix, so the value obtained from the matrix alone must always be subtracted from each sample of nanoparticles analysed. Thus, it is necessary to evaluate the matrix signal in each experiment and subtract it from the value obtained with the nanoparticles loaded with CHON.

2. Linearity

The proportionality of the amount of analyte present in the sample was evaluated, with respect to the signal obtained or the amount of titrant consumed [1,2]. Responses from samples containing different amounts of analyte are obtained from the test method. Linearity of the CHON assay was determined by analysis of three replicates of six concentrations of CHON. The acceptance criterion to determine linearity was the coefficient of determination (R^2) > 0.99.

- ***Titration method***

To titration method, the coefficient of determination (R^2) obtained was 0.9994, showing an excellent linear relation between the consumed volume of titrant and the amount of CHON present in the sample (**Figure S1**).

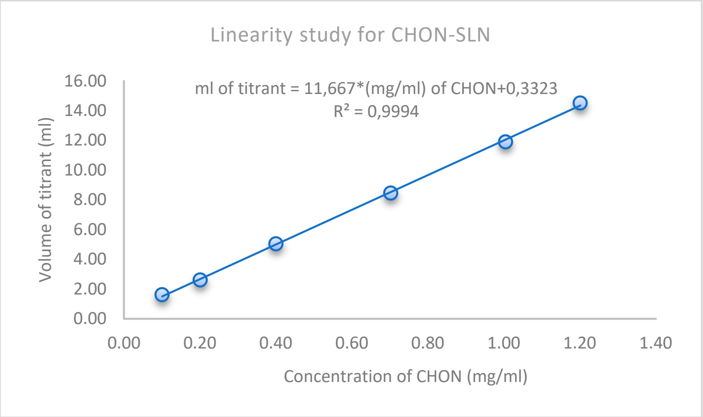

Figure S1: Linear regression curve for the assay of CHON in SLN.

Table S2: Linear regression results for the assay of linearity of CHON in SLN by titration method.

CHON concentration (mg/ml)	Average volume of titrant (ml)			
	**Sample 1**	**Sample 2**	**Sample 3**	**Average**
1.20	14.50	14.55	14.50	14.52
1.00	11.90	11.90	11.90	11.90
0.70	8.45	8.45	8.45	8.45
0.40	5.05	5.00	5.05	5.03
0.20	2.60	2.60	2.60	2.60
0.10	1.65	1.60	1.60	1.62

The values of the volume of titrant consumed increase linearly as the concentration of CHON increases (**Table S2**).

- ***UV-VIS spectrophotometric method***

For the spectrophotometric method, a high determination coefficient of 0.995 was obtained (**Figure S2**), fulfilling the linearity criterion of the method.

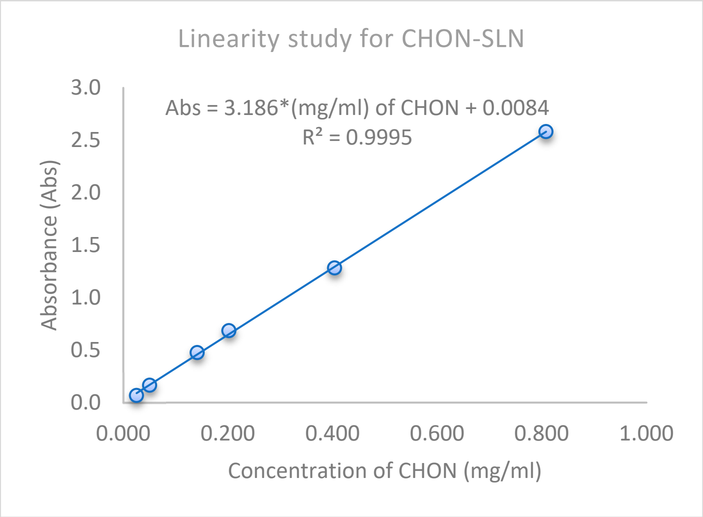

Figure S2: Linear regression curve for the assay of CHON in SLN.

Table S3: Linear regression results for the assay of linearity of CHON in SLN by UV-Vis spectrophotometric method.

**CHON concentration (mg/ml)**	**Absorbance (Abs)**			
	**Sample 1**	**Sample 2**	**Sample 3**	**Average**
0.808	2.541	2.620	2.582	2.581
0.404	1.284	1.281	1.279	1.281
0.202	0.700	0.667	0.685	0.684
0.141	0.477	0.471	0.474	0.474
0.051	0.163	0.163	0.163	0.163
0.025	0.064	0.067	0.064	0.065

The **Table S2** and **Table S3** show the individual values in the determination of the linear regression line of the studied methods. As the concentration of CHON in the enriched matrix increases (1.20, 1.00, 0.70, 0.40, 0.20, 0.10 mg/ml) both the amount consumed by the titrant in the volumetric titration or the spectrophotometric signal obtained increases.

3. Accuracy

"The accuracy of an analytical procedure expresses the closeness of agreement between the value which is accepted either as a conventional true value or an accepted reference value and the value found" [1,3].

The accuracy of a matrix enriched to (80 – 100 – 120) % of the final concentration of CHON (0.4 mg/ml) used in the nanoparticles was evaluated. The acceptance criteria were defined as the relative standard deviation (RSD %) of the measurement, with a maximum limit of 5.3 according to OAAC [4] and an average recovery between 90.0 to 110.0 %.

- ***Titration method***

The volumetric titration method meets the established accuracy criteria (

Table S4), since the RSD value was 1.4 less than 5.3 previously established and a recovery percentage of 93.3%, it is within the range 90.0% to 110.0%.

Table S4: Results of the accuracy determination for the study of CHON in SLN by titration method.

**Determination**	**Sample concentration (%)**	**Volume of titrant (ml)**	**CHON recovery (%)**
1	80	3.80	93.8
2	80	3.80	93.8
3	80	3.90	93.8
4	100	4.80	91.7
5	100	4.80	91.7
6	100	4.80	91.7
7	120	6.00	93.9
8	120	6.00	93.9
9	120	6.00	95.3
**Mean**	-	-	**93.3**
**SD**	-	-	**1.3**
**RSD**	-	-	**1.4**

- ***UV-VIS spectrophotometric method***

Table S5: Results of the accuracy determination for the study of CHON in SLN by spectrophotometric method.

**Determination**	**Sample concentration (%)**	**Absorbance (Abs)**	**CHON recovery (%)**
1	80	0.754	91.3
2	80	0.766	93.2
3	80	0.766	93.2
4	100	0.961	99.5
5	100	0.965	100.0
6	100	0.950	98.1
7	120	1.010	88.1
8	120	1.022	89.4
9	120	1.115	99.3
**Mean**	-	-	**94.7**
**SD**	-	-	**4.6**
**RSD**	-	-	**4.9**

The CHON recovery percentage in the matrix, with the spectrophotometric method, was higher than that obtained by the volumetric titration method, 94.7%, being also within the established range of 90.0% to 110.0% recovery (**Table S5**).

The variability found was higher with respect to the volumetric method, with an RSD of 4.9, even so it is less than 5.3. The variability in this method can be influenced, among other factors, by the volume of sulfuric acid required, since being a dense substance, the volume added can fluctuate and affect the final percentage recovered.
For both methods, it was fulfilled that the RSD value is below the proposed limit of 5.3, according to the AOAC regulations and within the set CHON recovery percentage [4].

4. Precision and intermediate precision

The consistency of the results obtained was evaluated, evaluating six samples at each level of analyte analyzed, as recommended in the ICH Q2 standards [1,3].

- ***Titration method***

To determine the precision of the method, two different titrants were analyzed on the same day, and intermediate precision was determined on different days with the same titrant.

Table S6: Results of intermediate precision determination for the study of CHON in SLN by titration method.

Determination	Sample concentration (%)	CHON recovery (%)	Mean	SD	RSD
T1-1	80	94.7	94.1	1.1	**1.1**
T1-2	80	92.7			
T1-3	80	94.7			
T1-4	80	94.7			
T1-5	80	92.7			
T1-6	80	94.7			
T2-1	80	96.1	94.2	0.9	**1.0**
T2-2	80	94.0			
T2-3	80	94.0			
T2-4	80	94.0			
T2-5	80	94.0			
T2-6	80	94.0			
D1-1	100	95.0	94.7	1.2	**1.3**
D1-2	100	95.0			
D1-3	100	96.6			
D1-4	100	95.0			
D1-5	100	93.4			
D1-6	100	93.4			
D2-1	100	93.5	93.9	0.7	**0.7**
D2-2	100	93.5			
D2-3	100	93.5			

D2-4	100	94.3			
D2-5	100	93.5			
D2-6	100	95.1			
Mean	-	**94.2**	-	-	-
SD	-	**1.0**	-	-	-
RSD	-	**1.0**	-	-	-

* D: day: T: Titrant

When determining the CHON recovery percentage in the Matrix (**Table S*6***), it was found that when determining the presence in 6 repeated measurements, for the 2 titrants used, the RSD value was 1.1 and 1.0 respectively and when doing the analysis with the same titrant on different days the RSD is 1.3 on day 1 and 0.7 on day 2. In general, the method is accurate, the RSD values per titrant group or per day were less than 5.3.

When evaluating the total data, taking the values obtained when using different titrants and on different days, the RSD is 1.0, which shows that it meets the intermediate precision of the method, since the global RSD value is less than 5.3.

- ***UV-VIS spectrophotometric method***

The precision of the spectrophotometric method was evaluated with 6 measurements of a sample and the intermediate precision when analyzing the samples on different days.

Table S7: Results of intermediate precision determination for the study of CHON in SLN by spectrophotometric method.

**Determination**	**Absorbance (Abs)**	**CHON recovery (%)**	**Mean**	**SD**	**RSD**
D1-1	0.496	92.0	95.1	3.5	3.7
D1-2	0.503	93.2			
D1-3	0.550	101.9			
D1-4	0.515	95.5			
D1-5	0.508	94.1			
D1-6	0.508	94.1			
D2-1	0.495	89.8	89.3	2.0	2.3
D2-2	0.494	89.6			
D2-3	0.499	90.5			
D2-4	0.488	88.6			
D2-5	0.500	90.7			
D2-6	0.467	85.0			
D2-7	0.508	90.9			
**Mean**	-	92.0	-	-	-
**SD**	-	4.0	-	-	-
**RSD**	-	4.4	-	-	-

Given the greater variability of the spectrophotometric method found in the determination of accuracy, there is also a higher RSD than that of the volumetric method. As is shown in**Table S7**, for day 1 the RSD was 3.7 and for day 2 it was 2.3 and for the intermediate precision, that involves the joint values found in the 2 days, it was 4.4. Even so, the UV-Vis method is accurate with RSD values less than 5.3.

5. Robustness

When evaluating the robustness of the method, small changes in its conditions were introduced to assess whether they are critical factors when applying the method [1].

- ***Titration method***

For the volumetric titration method, the effect of changing the titrant was evaluated, using another commercial brand (**Table S8**).

Table S8: Results of variation of the amount of volume of titrant in the determination of robustness for the study of CHON in SLN by titration method.

Determination	Titrant	Volume of titrant (ml)	CHON recovery (%)	Mean	SD	RSD
1	1	1.20	89.5	92.3	2.4	2.6
2	1	1.25	93.7			
3	1	1.25	93.7			
4	2	1.25	92.1	92.1	0.0	0.0
5	2	1.25	92.1			
6	2	1.25	92.1			
p Value	0.6579					

The t test (Nonparametric test), for the significance of differences among experimental groups ($p < 0.05$) was obtained by Mann Whitney test, and no significant differences were found, with a p value of 0.6579.
Therefore, the change proposed for the study of robustness in the volumetric method does not affect the analytical test for the determination of CHON in SLN.

- ***UV-VIS spectrophotometric method***

In the robustness study of the spectrophotometric method, specific changes were introduced, on the one hand, the variation of the wavelength, ±3 nm of the fixed working wavelength 520 nm, and on the other hand, changes in the volume (± 1 ml) of sulfuric acid added for the acid hydrolysis of glycosaminoglycans.

Table S9: Results of Wavelength (nm) variation in the robustness determination for the study of CHON in SLN by spectrophotometric method.

Determination	Sample concentration (%)	Wavelength (nm)	Absorbance (Abs)	CHON recovery (%)
1	120	520	1.726	96.0

2	120	520	1.725	96.0
3	120	520	1.714	95.3
4	120	517	1.724	96.3
5	120	517	1.722	96.2
6	120	517	1.713	95.7
7	120	523	1.719	95.8
8	120	523	1.717	95.6
9	120	523	1.706	95.0
10	100	520	1.322	87.6
11	100	520	1.435	95.3
12	100	520	1.417	94.1
13	100	517	1.411	94.0
14	100	517	1.323	87.9
15	100	517	1.440	96.0
16	100	523	1.315	87.3
17	100	523	1.425	94.8
18	100	523	1.408	93.7
19	80	520	1.136	93.6
20	80	520	1.108	91.2
21	80	520	1.136	93.6
22	80	517	1.142	94.3
23	80	517	1.112	91.7
24	80	517	1.141	94.2
25	80	523	1.125	92.9
26	80	523	1.099	90.7
27	80	523	1.124	92.9
**Mean**	_	_	_	93.6
**SD**	_	_	_	2.6
**RSD**	_	_	_	2.8

The data in **Table S9** show the results of the analysis of matrix samples spiked with different concentrations of CHON and evaluated at the different wavelengths. On average, a recovery of 93.6% and an RSD DE 2.8 was obtained. A One-way ANOVA test was performed for the significance of differences among multiple experimental groups was performed, not finding differences between groups, with an overall p value of 0.8078. After this, Tukey's Multiple Comparison Test was performed for the significance of differences among each pair of experimental groups ($p< 0.05$), and the results are presented below.

Table S10: Tukey's Multiple Comparison Test for changes in wavelength in the robustness study.

Groups evaluated	Significant? P < 0.05?	Summary
520 nm vs 517 nm	No	ns
520 nm vs 523 nm	No	ns

517 nm vs 523 nm	No	ns

ns: no significant differences

No significant differences were found in the different groups, analyzed by wavelength analyzed (**Table S10**). Therefore, a variation of ±3 nm in the wavelength does not affect the spectrophotometric analytical method.

In the study of the influence of the volume of sulfuric acid in the determination of the EE% of CHON in the nanoparticles, **Table S11** shows the results when varying the volumes of sulfuric acid.

Table S11: Results of absorbance variation in the robustness determination for the study of CHON in SLN by spectrophotometric method.

Determination	Volume of sulfuric acid (ml)	Absorbance (Abs)	CHON recovery (%)	Mean	SD	RSD
1	5	0.652	93.7	93.6	2.2	2.3
2	5	0.666	95.8			
3	5	0.637	91.4			
4	4	0.537	76.4	76.5	3.3	4.3
5	4	0.560	79.8			
6	4	0.516	73.2			
7	6	0.453	63.7	63.6	2.0	3.2
8	6	0.465	65.5			
9	6	0.438	61.5			

The One-way ANOVA test was performed due to the significant differences among multiple experimental groups, finding discrepancies in the global analysis between groups, with a value of $p < 0.0001$. This was followed by Bonferroni's Multiple Comparison Test for the significance of differences among each pair of experimental groups ($p < 0.05$), and the results are presented below.

Table *S12:* *Bonferroni's Multiple Comparison Test for changes in the volume of sulfuric acid used in the robustness study.*

Groups evaluated	Significant? P < 0.05?	Summary
H_2SO_4 (5.0 mL) vs H_2SO_4 (4.0 mL)	Yes	***
H_2SO_4 (5.0 mL) vs H_2SO_4 (6.0 mL)	Yes	***
H_2SO_4 (4.0 mL) vs H_2SO_4 (6.0 mL)	Yes	**

*p< 0.05, **p< 0.01 and ***p< 0.001

Since there are statistically significant differences between the recovery percentages according to the volume of acid added (**Table S12**), and very low results (76.5 % and 63.6 %) are obtained using 4.0 or 6.0 ml respectively, it is necessary to be sure to add the 5.0 ml volume.

Table S13: Summary of the results obtained in the validation of the analytical methods for the determination of the EE% of CHON in the SLN.

**Parameter**	**Method Title**	**UV-Vis method**
Specificity / Selectivity<br>No reaction with the matrix / % discrepancy ≤ ± 5%	White does not react with titrant	Discrepancy (%) 21.09<br>Student'st test $p < 0.0001$
Linearity ($r^2 > 0.99$)	0.9994	0.9995
Precision (%CV ≤ 5.3)	1,3	3.7
Precision intermediate (%CV ≤ 5.3)	1.1	3.0
Accuracy–SLN(%)<br>RSD < 5.3<br>Recovery between 90.0 and 110.0%	1.4<br>93.3	4.9<br>94.7
Robustness<br>Statistical test	Changes do not affect the results	Different volumes of sulfuric acid produce significant changes in the result.

In general, according with **Table S13**, both methods met the established validation criteria. The volumetric method presents less variation in the means compared to the spectrophotometric method; the latter being influenced by the precision in the addition of sulfuric acid. The spectrophotometric method achieved a higher CHON recovery percentage and always requires the measurement and correction of the matrix signal.

References

[1] ICH Harmonised Tripartite Guideline. Validation of Analytical Procedures: Text And Methodology Q2(R1) [Internet]. 2005 [cited 2022 Sep 16]. Available from: https://somatek.com/wp-content/uploads/2014/06/sk140605h.pdf

[2] USP. <1225> Validation of Compendial Procedures. In: USP 40/NF 35, United States Pharmacopeia. 40th ed., National Formulary. 35th ed. 35th ed. Rockville, MD: The United States Pharmacopeia Convention; United Book Press, Inc; 2017.

[3] Jenkins D, Diallo C, Bethea E, Kaale E, Layloff T. Method Validation Approaches for Pharmaceutical Assessments – Highlights with High Performance Thin Layer Chromatographic (HPTLC) Techniques. Calibration and Validation of Analytical Methods - A Sampling of Current Approaches. InTech; 2018.
[4] AOAC International Officers and Committees. J AOAC Int. 1993 Jan 1;76(1):223–50.
